# Sarecycline Demonstrated Reduced Activity Compared to Minocycline against Microbial Species Representing Human Gastrointestinal Microbiota

**DOI:** 10.3390/antibiotics11030324

**Published:** 2022-02-28

**Authors:** Mahmoud A. Ghannoum, Lisa Long, Christopher G. Bunick, James Q. Del Rosso, Ahmed Gamal, Stephen K. Tyring, Thomas S. McCormick, Ayman Grada

**Affiliations:** 1 Center for Medical Mycology, Integrated Microbiome Core, Department of Dermatology, Case Western Reserve University, Cleveland, OH 44106, USA; lal11@case.edu (L.L.); axg1117@case.edu (A.G.); tsm4@case.edu (T.S.M.); 2 Department of Dermatology, University Hospitals Cleveland Medical Center, Cleveland, OH 44106, USA; 3 Department of Dermatology, Yale University School of Medicine, New Haven, CT 06520, USA; christopher.bunick@yale.edu; 4 JDR Dermatology Research/Thomas Dermatology, Las Vegas, NV 89148, USA; jqdelrosso@yahoo.com; 5 Center for Clinical Studies, Webster, TX 77598, USA; styring@ccstexas.com; 6 Department of Dermatology, University of Texas Health Science Center, Houston, TX 77030, USA; 7 R&D and Medical Affairs, Almirall LLC, Malvern, PA 19355, USA; grada@bu.edu

**Keywords:** sarecycline, minocycline, antibiotics, antimicrobial activity, gut, microbiome, acne vulgaris

## Abstract

Prolonged use of broad-spectrum tetracycline antibiotics such as minocycline and doxycycline may significantly alter the gut and skin microbiome leading to dysbiosis. Sarecycline, a narrow-spectrum tetracycline-class antibiotic used for acne treatment, is hypothesized to have minimal impact on the gastrointestinal tract microbiota. We evaluated the effect of sarecycline compared to minocycline against a panel of microorganisms that reflect the diversity of the gut microbiome using in vitro minimum inhibitory concentration (MIC) and time-kill kinetic assays. Compared to minocycline, sarecycline showed less antimicrobial activity indicated by higher MIC against 10 of 12 isolates from the Bacteroidetes phylum, three out of four isolates from Actinobacteria phylum, and five of seven isolates from the Firmicutes phylum, with significantly higher MIC values against *Propionibacterium freudenreichii* (≥3 dilutions). In time-kill assays, sarecycline demonstrated significantly less activity against *Escherichia coli* compared to minocycline at all time-points (*p* < 0.05). Moreover, sarecycline was significantly less effective in inhibiting *Candida tropicalis* compared to minocycline following 20- and 22-h exposure. Furthermore, sarecycline showed significantly less activity against *Lactobacillus paracasei* (recently renamed as *Lacticaseibacillus paracasei* subsp. *paracasei*) (*p* = 0.002) and *Bifidobacterium adolescentis* at 48 h (*p* = 0.042), when compared to minocycline. Overall, sarecycline demonstrated reduced antimicrobial activity against 79% of the tested gut microorganisms, suggesting that it is less disruptive to gut microbiota compared with minocycline. Further in vivo testing is warranted.

## 1. Introduction

The composition of the human microbiome varies across body sites with the greatest concentration and diversity of microorganisms found in the gastrointestinal tract [1]. Recent studies have established that the gut microbiota plays an important role in the biology of health and disease [2]. Thus, maintaining balance of the microbial communities (e.g., bacteria and fungi) is critical. Medications, both antibiotics (mostly broad-spectrum antibiotics) and non-antibiotics (e.g., immunosuppressive drugs) have been reported to have unintended effects on the gut microbial communities leading to an imbalance in the composition of commensal gut organisms, often called dysbiosis [3,4,5,6].

Intestinal dysbiosis has been shown to cause profound inflammation, which is associated with numerous chronic diseases. For example, individuals with type 2 diabetes were found to have increased levels of *Akkermansia muciniphila* and reduced *Roseburia* and *Lactobacillus* species in their gut [7,8,9,10,11]. Additionally, several microorganisms were reported to be reduced in obese people such as *Akkermansia muciniphila*, *Anaerotruncus colihominis*, *Butyrivibrio crossotus*, *Methanobrevibacter smithii*, *Alistipes*, and *Barnesiella* [12,13,14,15,16,17]. Furthermore, reduction of *Oxalobacter formigenes* species were linked to kidney stone formation [18,19]. Another important example is the microbial dysbiosis that has been linked to several manifestations observed in inflammatory bowel disease (IBD) and atopic dermatitis patients [20,21,22,23]. Interestingly, treatment of mice with broad-spectrum antibiotics caused severe perturbation of the gut microbiota and acceleration of breast tumor growth [24]. Moreover, although no causal relationship has been definitively established, the use of doxycycline in acne vulgaris patients was found to be associated with a 2.25-fold greater risk for developing Crohn’s disease [25]. For this reason, it is important to consider the potential effects an antibiotic may have on the gut microbiota.

Gut dysbiosis effect extends beyond the digestive system and can alter the microbiota present in other body sites including the skin through what is known as gut-skin-axis [26]. In a study by Thompson et al., minocycline caused significant dysbiosis in the skin and gastrointestinal tract of acne patients, including impacting many probiotic species [27]. In terms of the cutaneous microbiome, there was depletion in *Staphylococcus epidermidis* and *Prevotella nigrescens* in treated acne patients. *S. epidermidis* is a Gram-positive bacterium that colonizes normal human skin and was shown to inhibit growth of *Cutibacterium acnes* (formerly known as *Probpionibacterium acnes*) in in vitro studies, which is an anaerobic bacterium that plays a major role in the pathogenesis of acne [28]. In terms of the gut microbiome, minocycline-treated patients had reduction in the abundance of many probiotic species including *Lactobacillus salivarius*, *Bifidobacterium adolescentis*, *Bifidobacterium pseudolongum*, and *Bifidobacterium breve* [27]. These probiotic bacteria are reported to have antidepressant effects [29,30], modulate the immune response [31], and reduce harmful gut colonization by direct inhibition of competing pathogens.

Sarecycline is the first narrow-spectrum drug in the tetracycline class of antibiotics, developed to treat acne vulgaris [32]. Zhanel et al., showed that sarecycline was 16- to 32-fold less active than broad-spectrum tetracyclines against aerobic Gram-negative enteric bacilli commonly found in the human gastrointestinal tract [33]. Sarecycline was also less effective against *Escherichia coli* using an in vivo murine septicemia model, when compared to doxycycline [34]. Furthermore, sarecycline was four- to eight-fold less active than doxycycline against representative anaerobic bacteria that also comprise the human intestinal microbiota. Based on this data, we hypothesized that sarecycline may have less impact on the gut microbiota compared to other broad-spectrum antibiotics.

The aim of this study was to evaluate the effect of sarecycline compared to minocycline on representative microbiota (both bacteria and fungi) commonly found in the human gastrointestinal tract using an in-vitro approach. Importantly, although antibiotics are known to have minimal impact on fungi, our data showed that minocycline have inhibitory activity against *Candida* [35]. Based on this, we include 4 different *Candida* species in our study comparing minocycline and sarecycline.

## 2. Results

### 2.1. Effect of Sarecycline Compared to Minocycline on Gut Microbiota In Vitro

Table 1 shows the MICs for sarecycline and minocycline against the isolates tested (*n* = 28). Overall, sarecycline demonstrated less in vitro activity against most isolates tested compared to minocycline which is indicated by the higher MIC values for sarecycline as determined by antibiotic susceptibility test (i.e., higher MIC value = less inhibitory effect).

Specifically, against Actinobacteria phylum, sarecycline had a higher MIC range compared to minocycline (1–8 µg/mL vs. 0.5–1 µg/mL, respectively) with greatest MIC fold difference observed against *Propionibacterium freudenreichii* (8 vs. 0.25 µg/mL, respectively). *Propionibacterium freudenreichii* is a known probiotic strain that produces beneficial products including short chain fatty acids, folate and cobalamin vitamins [36].

Additionally, sarecycline exhibited less antibacterial activity compared to minocycline against isolates belonging to the Bacteroidetes phylum (*n* = 12). In this regard, sarecycline had MIC range of 0.06–>8 µg/mL which was higher than the range observed with minocycline (0.016–8 µg/mL). Notably, the biggest MIC fold difference between sarecycline and minocycline was observed against *Bacteroides vulgatus* (0.125 vs. 0.016 µg/mL, respectively). *Bacteroides vulgatus* is a bacterium that was recently reported to be reduced in patients with atherosclerosis. Furthermore, gavage with this organism was shown to reduce the formation of atherosclerotic lesions in atherosclerosis-prone mice [37].

Against the Firmicutes phylum isolates (*n* = 7), sarecycline had lower activity compared to minocycline which is demonstrated by higher MIC range of 0.25–>8 µg/mL compared to 0.06–4 µg/mL for minocycline. Moreover, sarecycline showed significantly higher MICs, when compared to minocycline, against *Clostridium bolteae* (a bacterium reported to play a role in induction of T regulatory cells in mice colon) [38], and *Erysipelatoclostridium ramosum* (previously known as *Clostridium ramosum*) which was shown to have a regulatory effect on enterochromaffin cell development and serotonin release in mice [39].

Against the yeast isolates tested (*n* = 4), sarecycline tended to have less antifungal activity compared to minocycline against *Candida albicans* and *C. parapsilosis*, albeit this was not significant. Thus, testing against a larger panel of yeast should be undertaken.

### 2.2. Effect of Sarecycline and Minocycline on Microbial Growth

#### 2.2.1. Aerobic Species

**Figure 1:**
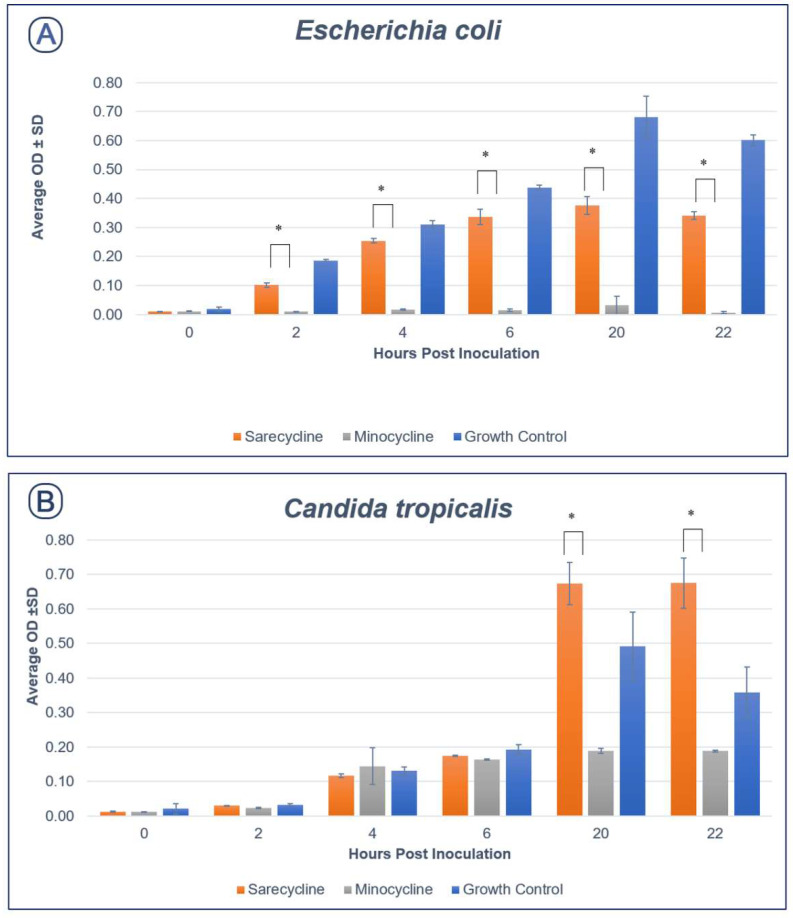
Histograms for *Escherichia* *coli* (**A**) and *Candida tropicalis* (**B**) in the presence of sarecycline and minocycline as measure by optical density (OD). * Sarecycline showed significantly less antimicrobial activity when compared to minocycline, *p*-value of <0.05.

Using time-kill assay, sarecycline exhibited significantly less activity against *Escherichia* *coli* compared to minocycline at all time points (*p* < 0.05). Similarly, sarecycline was significantly less effective in inhibiting *C. tropicalis* compared to minocycline at 20- and 22-h post-exposure (*p* < 0.05) (Figure 1).

#### 2.2.2. Anaerobic Species

**Figure 2:**
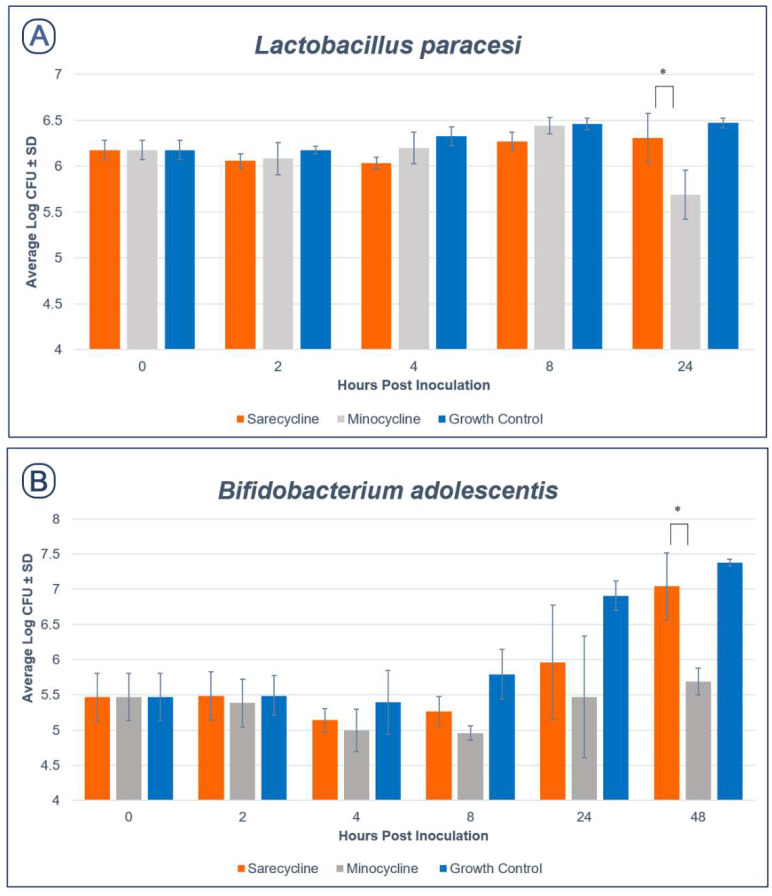
Growth Curve Data for *Lactobacillus paracasei* (**A**) and *Bifidobacterium adolescentis* (**B**) in the presence of sarecycline and minocycline. * Sarecycline showed significantly less antimicrobial activity when compared to minocycline, *p*-value of <0.05.

Growth kill curves for *Lactobacillus paracasei* and *Bifidobacterium adolescentis* in the presence of sarecycline and minocycline are shown in Figure 2. As seen in Figure 2A, sarecycline showed significantly less activity against *Lactobacillus paracasei* compared to minocycline at 24 h of growth (*p* = 0.002). Moreover, sarecycline showed significantly less activity against *Bifidobacterium adolescentis* compared to minocycline after 48 h of growth (*p* = 0.042, see Figure 2B).

## 3. Discussion

We compared the activity of sarecycline vs. minocycline against representative microbes commonly found in the normal human gut using in vitro susceptibility testing. Sarecycline demonstrated higher MIC values against 22 out of 28 isolates tested including *Escherichia* *coli IAI1*, *Bacteroides* *caccae*, *Bacteroides* *vulgatus*, *Clostridium* *bolteae*, *Clostridium* *ramosum*, *Candida albicans*, and *Candida parapsilosis*. This data suggests that sarecycline may have less damaging effect on the gut microbiome compared to minocycline. This was further supported by the data obtained using time-kill assays in which sarecycline demonstrated less activity in inhibiting the growth of representative bacteria and fungi compared to minocycline. Overall, sarecycline showed decreased in vitro activity compared to minocycline against 79% of the tested gut microbiome.

Our results are consistent with previous studies in which sarecycline demonstrated reduced activity against enteric Gram-negative bacteria [33]. Zhanel et al., compared the activity of sarecycline to tetracycline, doxycycline and minocycline and showed that it was 16- to 32-fold less active against aerobic Gram-negative bacilli including 33 isolates of *Escherichia* *coli,* with MIC_50_ (minimum inhibitory concentration that inhibit 50% of the strains tested) of 16 µg/mL, whereas the MIC_50_ for tetracycline, doxycycline, and minocycline were 2, 2, and 1 µg/mL, respectively [33]. Sarecycline activity was also compared to the other tetracycline-class antibiotics against 389 contemporary clinical isolates from 10 members of the Enterobacteriaceae and the normal flora found in the human intestinal tract. This data showed that sarecycline was the least active antibiotic against the tested isolates with an MIC range of 1 to >256 µg/mL; however, sarecycline showed equivalent activity to the comparators against Gram-positive cocci including *Staphylococcus aureus* [33].

In our study, sarecycline demonstrated less activity compared to minocycline against isolates from the Bacteroidetes phylum including a number of beneficial strains such as *Bacteroides fragilis nontoxigenic* and *Bacteroides vulgatus.* *Bacteroides fragilis nontoxigenic* is a member of the gut microbiota that was recently proposed to be a potential probiotic because of its protective function against colitis using the CD4 + CD45Rb transfer model of experimental colitis [40]. This protection was conferred by inducing anti-inflammatory functions of regulatory T cells and altering the pro-inflammatory cytokines that play a role in the disease using polysaccharide A [41,42,43], as well as correction of intestinal permeability and improving symptoms of autism in offspring of maternal immune activation mice [44]. Interestingly, reports of *Bacteroides fragilis* levels in IBD patients compared with healthy controls were variable hence further studies are needed to define the role of *Bacteroides fragilis* in IBD [45,46]. Additionally, Yoshida et al., has reported an association between reduction of *Bacteroides vulgatus* and atherosclerosis [37]. In order to investigate this observation, the study group treated atherosclerosis-prone mice with live *Bacteroides vulgatus* using oral gavage five times per week for 10 weeks. Interestingly, a significant reduction in the atherosclerotic lesion size in the aortic root was observed in the treated group compared to controls (i.e., untreated). This data suggests that use of a narrower spectrum antibiotics, such as sarecycline, may help in preserving these beneficial microorganisms. However, in vivo testing as well as clinical trials to determine the effect of this antibiotic on the human gut microbiome warranted.

*Firmicutes* comprise the majority of the intestinal microbiota [47]. Furthermore they are known for their ability to ferment fibers and produce short chain fatty acids (SCFAs), mainly butyrate [48,49], that play an important role in small intestinal cell proliferation and regulation of epithelial gene expression [50,51], integrity of epithelial barrier [52,53,54,55,56], act as the main energy source of colonocytes [57,58], and anti-inflammatory effects [59]. Our data showed that sarecycline had less activity compared to minocycline against isolates belonging to this phylum. The higher activity of minocycline observed in our study is in agreement with recent studies showing that doxycycline and minocycline affect the relative abundance (percentage of a specific organism within the entire microbiome) of the gut microbiome [3,60] as well as increase organisms possessing genes associated with antibiotic resistance which may be explained by the broad-spectrum activity of these agents [5,61]. Thus, we suggest that the use of antibiotic agents that demonstrate less inhibitory activity against micrbail community that resides in the gut would be helpful in keeping the integrity of this microbial population preventing gut dysbiosis.

Gut dysbiosis, an imbalance between the types of microorganisms that inhabit a person’s body, has been associated with immune dysregulation, alteration of Th-1 cell response and up-regulation of gene expression of pro-inflammatory cytokines including IFN-γ, IL-17A, TNF-α, and IL-1β [62]. Furthermore, it is has been also reported as a potential cause for disruption of the gut mucosal barriers leading to a condition known as leaky gut [4,63], which is characterized by increased gut permeability and translocation of intestinal microbes into the blood circulation [64,65,66]. Use of broad spectrum antimicrobial would facilitated these events which, indirectly, may increase the risk for a variety of diseases; including IBD [67,68], celiac diseases [69], and systemic lupus erythematosus [70,71].

In the current study *Bifidobacterium adolescentis* demonstrated equivalent in vitro susceptibility, as measured by MIC, to sarecycline and minocycline. However, using growth-kill kinetic assays showed that sarecycline was significantly less active against *Bifidobacterium adolescentis* compared to minocycline after 48 h of growth. This may be explained by the ability of minocycline to demonstrate a combined time-dependent and concentration-dependent killing effect with extended post-antibiotic effect [72,73]. A similar observation has been reported in a study by Bowker et al., in which minocycline exhibited both combined time-dependent and concentration-dependent killing effects against *Staphylococcus aureus* in an in vitro pharmacokinetic model [74]. This might indicate that although minocycline and sarecycline showed equivalent in vitro MIC values against *Bifidobacterium adolescentis*, minocycline, unlike sarecycline, can cause greater alteration to the gut microbiome due to its post-antibiotic effect.

Use of broad-spectrum antibiotics may result, unintentionally, in elimination of beneficial microbiota which in turn facilitate the tissue colonization by opportunistic microbial pathogens. In this regard, doxycycline and minocycline were reported in several studies to be associated with a number of *Candida*-related illnesses including vulvovaginal candidiasis, vulvovaginal mycotic infection [75]. In contrast, in published clinical trials, sarecycline demonstrated low incidence of vulvovaginal mycotic infection (0.8%) and vulvovaginal candidiasis (0.6%) [76,77]. Furthermore, the use of broad-spectrum antibiotics has been linked to the emergence of *Candida*-resistant strains which might cause life-threatening bloodstream infection in susceptible patients [78]. Similar findings have been reported with doxycycline and minocycline [35].

Another critical observation in our study is that sarecycline showed less activity against *Lactobacilli* compared to minocycline. *Lactobacilli* play an important role in blocking yeast adhesion to the epithelium while, at the same time, producing inhibitory substances (e.g., volatile short chain fatty acids and secondary bile acids) that can reduce the ability of *Candida* to form hyphae and invasion [79]. These observations are further supported by studies showing that germfree mice being more susceptible to *Candida* colonization [80]. Additionally, colonization of the gut with *Candida albicans* was shown to be reduced in mice treated with *Lactobacillus* probiotic strains compared to the untreated mice [80]. This bidirectional antagonistic relationship between *Candida* and *Lactobacilli* in which the presence of one inhibits growth of the other has been further investigated in literature [81]. Thus, based on our results, the use of antibiotics that are less damaging to the microbiome such as sarecycline may help in maintaining the balance of the microbiota and consequently reducing the incidence of undesired health effects.

## 4. Materials and Methods

### 4.1. Representative Gut Bacterial and Fungal Strains

To evaluate the activity of sarecycline compared to minocycline against organisms that normally reside in the gut, we selected microorganisms intended to reflect the diversity of the human gut microbiome sourced from the Deutsche Sammlung von Mikroorganismen und Zellkulturen (DSMZ), the American Type Culture Collection (ATCC), and the Center for Medical Mycology culture collection (CMM) (Table 2) [82]. The efficacy of sarecycline and comparators against these organisms was determined using susceptibility testing and time-kill assays [83].

### 4.2. Antimicrobial Susceptibility Testing

#### 4.2.1. Anaerobic Bacteria

Minimum inhibitory concentration (MIC) testing was performed for anaerobic bacteria using an anaerobic chamber following a modified Clinical Laboratory Standards Institute (CLSI) M11-A7 agar dilution methodology [83]. Bacteria were grown on Brucella Blood Agar plates (Remel Microbiology Products, Columbus, OH, USA) supplemented with Hemin (Sigma-Aldrich, St. Louis, MO, USA) and vitamin K (Sigma-Aldrich, St. Louis, MO, USA) and infused with various concentrations of sarecycline or minocycline (0.016–8 µg/mL). Infused agar was inoculated with 2 µL of 1 to 2 × 10^8^ colony forming units (CFUs)/mL and incubated at 37 °C for 48 h in an anaerobic atmosphere. The lowest concentration of the antimicrobial agent that resulted in a visually evaluated inhibition of growth was recorded and MIC evaluated.

#### 4.2.2. Yeasts

*Candida* isolates were tested using a modified CLSI M27-A4 broth microdilution method at a range of 0.125–64 µg/mL. RPMI 1640 broth was inoculated with 0.5 to 2.5 × 10^3^ CFUs/mL, and incubated at 37 °C for 24 h. The lowest concentration of the antimicrobial agent that resulted in 50% growth inhibition when compared to the untreated growth control was recorded.

### 4.3. Aerobic Growth Curve Conditions

To compare the effect of sarecycline and minocycline on growth kinetics, we selected *Escherichia* *coli* and *Candida tropicalis* as representative bacterial and yeast organisms, respectively. Strains were grown in culture media specific to species as described previously [84]. Brain Heart Infusion (BHI) broth was used to culture *Escherichia* *coli*. To evaluate the effect of sarecycline and comparators against *Candida* species, yeast cells were grown in buffered RPMI-1640. All experiments were performed at 37 °C. Strains were expanded by overnight culture twice.

The concentration of sarecycline in each well tested was 20 μM, which is within the expected concentration of drug reported in the gut previously [85]. The antibiotics were dissolved in 2% dimethyl sulfoxide (DMSO) at twice the desired concentration. The starting inoculum was standardized spectrophotometrically at an optical density (OD) of 0.01 measured at a wavelength (λ) of 528 nm. At different time points (2-, 4-, 6-, 20- and 22-h post inoculation), an aliquot was removed and optical density was measured. Next, time-kill curves were constructed.

### 4.4. Anaerobic Growth Curve Conditions

To compare the effect of sarecycline and minocycline on growth kinetics, we selected *Lactobacillus paracasei* and *Bifidobacterium adolescentis* as representative anaerobic bacteria that colonize the gut. BHI broth was inoculated with 2 µL of 1 to 2 × 10^8^ CFUs/mL and incubated at 37 °C. Strains were grown in the presence of 0.5× the MIC of sarecycline and minocycline. At various timepoints (0, 2-, 4-, 8-, 24-, and 48-h post-inoculation) samples were taken and CFUs/mL assessed. Next, time-kill curves were constructed.

Differences in the mean Log CFU/mL were compared across groups using a one-way ANOVA with a post-hoc Bonferroni (IBM, SPSS ver 27.0). A *p*-value of <0.05 was considered statistically significant.

## 5. Conclusions

Dermatologists prescribe more oral antibiotic courses per clinician than any other specialty, and many of these courses of antibiotics are prescribed for several months in duration [86]. The prolonged and intermittent use of broad-spectrum antibiotics has been associated with the development of antimicrobial resistance and permanent perturbation of the gut microbiome [87,88]. Recent advances in understanding the role of the microbiome in health and disease underscore the importance of antibiotic stewardship in dermatology. One way to overcome this issue could be the use of narrow-spectrum antibiotics which are less likely to cause gut dysbiosis. In this regard, our results indicate that sarecycline has lower in vitro activity compared to minocycline against the most common microorganisms that inhibit the gastrointestinal tract. Furthermore, such a narrow spectrum activity could be a viable treatment option for patients with moderate-to-severe acne vulgaris who may require prolonged systemic antibiotic treatment. However, more studies are needed to confirm its activity in in vivo settings as well as human subjects.

## Figures and Tables

**Table 1:** Susceptibility testing results for sarecycline and minocycline against the strains tested in µg/mL (*n* = 28).

Phylum	Genus	Species	Sarecycline	Minocycline	MIC Fold Difference
Actinobacteria	*Bifidobacterium*	*Bifidobacterium adolescentis*	1	1	1
Actinobacteria	*Collinsella*	*Collinsella aerofaciens*	1	0.5	2
Actinobacteria	*Eggerthella*	*Eggerthella lenta*	1	0.5	2
Actinobacteria	*Actinomycetales*	*Propionibacterium freudenreichii*	8	1	8
Bacteroidetes	*Bacteroides*	*Bacteroides caccae*	8	0.25	32
Bacteroidetes	*Bacteroides*	*Bacteroides fragilis enterotoxigenic (ET)*	2	4	0.5
Bacteroidetes	*Bacteroides*	*Bacteroides fragilis nontoxigenic*	1	0.25	4
Bacteroidetes	*Bacteroides*	*Bacteroides ovatus*	0.5	0.5	1
Bacteroidetes	*Bacteroides*	*Bacteroides thetaiotaomicron*	0.25	0.125	2
Bacteroidetes	*Bacteroides*	*Bacteroides uniformis*	2	0.5	4
Bacteroidetes	*Bacteroides*	*Bacteroides vulgatus*	0.125	0.016	7.8
Bacteroidetes	*Bacteroides*	*Bacteroides xylanisolvens*	1	0.25	4
Bacteroidetes	*Bacteroides*	*Bifidobacterium subtile Biavati*	>8	8	ND *
Bacteroidetes	*Odoribacter*	*Odoribacter splanchnicus*	8	4	2
Bacteroidetes	*Parabacteroides*	*Parabacteroides distasonis*	8	2	4
Bacteroidetes	*Parabacteroides*	*Parabacteroides merdae*	0.06	0.016	3.8
Firmicutes	*Blautia*	*Blautia obeum*	1	0.5	2
Firmicutes	*Clostridium*	*Clostridium bolteae*	4	0.5	8
Firmicutes	*Clostridium*	*Erysipelatoclostridium ramosum*	2	0.06	33.3
Firmicutes	*Clostridium*	*Clostridium saccharolyticum*	2	2	1
Firmicutes	*Dorea*	*Dorea formicigenerans*	0.25	0.06	4.2
Firmicutes	*Eubacterium*	*Eubacterium eligens*	>8	4	ND *
Firmicutes	*Lactobacillus*	*Lactobacillus paracasei*	1	0.25	4
Proteobacteria	*Escherichia*	*Escherichia coli IAI1*	16	8	2
Ascomycota	*Candida*	*Candida albicans*	32	16	2
Ascomycota	*Candida*	*Candida glabrata*	32	32	1
Ascomycota	*Candida*	*Candida parapsilosis*	32	16	2
Ascomycota	*Candida*	*Candida tropicalis*	16	16	1

* ND—Not Determined.

**Table 2:** Representative microbial species commonly found in the human gastrointestinal tract.

Phylum	Genus	Species	Source
Bacteroidetes	*Bacteroides*	*Bacteroides vulgatus*	DSMZ 1447
Bacteroidetes	*Bacteroides*	*Bacteroides uniformis*	DSMZ 6597
Bacteroidetes	*Bacteroides*	*Bacteroides fragilis nontoxigenic*	ATCC 43858
Bacteroidetes	*Bacteroides*	*Bacteroides thetaiotaomicron*	DSMZ 2079
Bacteroidetes	*Bacteroides*	*Bifidobacterium subtile Biavati*	ATCC 27537
Firmicutes	*Clostridium*	*Clostridium ramosum*	DSMZ 1402
Actinobacteria	*Bifidobacterium*	*Bifidobacterium adolescentis*	DSMZ 20083
Actinobacteria	*Eggerthella*	*Eggerthella lenta*	DSMZ 2243
Firmicutes	*Clostridium*	*Clostridium bolteae*	DSMZ 15670
Bacteroidetes	*Bacteroides*	*Bacteroides fragilis enterotoxigenic (ET)*	ATCC 43860
Firmicutes	*Clostridium*	*Clostridium saccharolyticum*	DSMZ 2544
Firmicutes	*Lactobacillus*	*Lactobacillus paracasei*	DSMZ 5622
Bacteroidetes	*Bacteroides*	*Bacteroides caccae*	DSMZ 19024
Bacteroidetes	*Bacteroides*	*Bacteroides ovatus*	DSMZ 1896
Bacteroidetes	*Bacteroides*	*Bacteroides xylanisolvens*	DSMZ 18836
Firmicutes	*Blautia*	*Blautia obeum*	DSMZ 25238
Bacteroidetes	*Parabacteroides*	*Parabacteroides merdae*	DSMZ 19495
Actinobacteria	*Collinsella*	*Collinsella aerofaciens*	DSMZ 3979
Actinobacteria	*Actinomycetales*	*Propionibacterium freudenreichii*	CMM
Bacteroidetes	*Parabacteroides*	*Parabacteroides distasonis*	DSMZ 20701
Firmicutes	*Eubacterium*	*Eubacterium eligens*	DSMZ 3376
Firmicutes	*Dorea*	*Dorea formicigenerans*	DSMZ 3992
Proteobacteria	*Escherichia*	*Escherichia coli IAI1*	CMM
Bacteroidetes	*Odoribacter*	*Odoribacter splanchnicus*	DSMZ 20712
Ascomycota	*Candida*	*Candida albicans*	CMM
Ascomycota	*Candida*	*Candida tropicalis*	CMM
Ascomycota	*Candida*	*Candida parapsilosis*	CMM
Ascomycota	*Candida*	*Candida glabrata*	CMM
